# Post-warming culture of human vitrified blastocysts with prolactin improves trophoblast outgrowth

**DOI:** 10.1186/s12958-023-01062-0

**Published:** 2023-01-18

**Authors:** Kenji Ezoe, Nanoha Fujiwara, Tetsuya Miki, Keiichi Kato

**Affiliations:** Kato Ladies Clinic, 7-20-3 Nishishinjuku, Shinjuku-ku, Tokyo 160-0023 Japan

**Keywords:** Blastocyst, Prolactin, Recovery culture, Vitrification, Trophoblast migration

## Abstract

**Background:**

Human embryos express the prolactin (PRL) receptor at the morula and blastocyst stages. Treatment with PRL from cleavage to the blastocyst stage improves blastocyst outgrowth on fibronectin-coated dishes. However, whether post-warming PRL treatment of blastocysts cultured without PRL could improve outgrowth competence remains unknown. Furthermore, the optimal time for post-warming PRL treatment remains to be ascertained. This study investigated the effects of PRL treatment during recovery culture on human blastocyst outgrowth and its related genes.

**Methods:**

In total, 374 discarded vitrified blastocysts were randomly allocated to two groups, to be cultured with (*n* = 208) or without PRL (control; *n* = 166) for 120 min for recovery, and then plated on fibronectin-coated dishes. The expression level of PRL-interacting genes, blastocyst adhesion rate, outgrowth area, distance of trophoblast migration, and outgrowth degeneration were examined.

**Results:**

The mRNA expression of ezrin, radixin, and moesin, which regulate cell adhesion and invasion by controlling actin reorganization during epithelial-to-mesenchymal transition (EMT), was stimulated by PRL treatment for 120 min. The expression of EMT-related genes, transforming growth factor β1, snail1, and twist1 was also promoted following treatment with PRL for 120 min. PRL-treated blastocysts also exhibited augmented expression of cadherin 2 and transcriptional repression of cadherin 1. Higher mRNA expression of integrin-based focal adhesion-related genes, ITGA5 and ITGB1, was observed after treatment with PRL for 120 min than in the non- and shorter-treatment groups. PRL treatment for 120 min did not alter the rate of blastocyst adhesion to fibronectin-coated dishes 96 h after the outgrowth culture assay. However, multiple linear regression analysis revealed that the outgrowth area was significantly increased in PRL-treated blastocysts. The migration distance of trophoblast cells was significantly increased and degeneration rate was significantly decreased after PRL treatment. Furthermore, a more beneficial effect of PRL treatment on blastocyst outgrowth was observed when the blastocysts were vitrified on day 5 than when they were vitrified on day 6.

**Conclusions:**

Post-warming culture of human vitrified blastocysts with PRL for 120 min promoted trophoblast outgrowth in vitrified human blastocysts. Furthermore, PRL treatment may reduce outgrowth degeneration by increasing resistance to apoptosis during trophoblast migration.

**Supplementary Information:**

The online version contains supplementary material available at 10.1186/s12958-023-01062-0.

## Background

Implantation is the first physical as well as physiological contact between an implantation-competent blastocyst and the receptive uterus [1]. The embryo–uterine cross-talk orchestrates the molecular dialogue and steers the establishment of successful implantation [2]. During the pre-implantation period, the endometrium is programmed to secrete various kinds of molecules with autocrine and paracrine functions, and embryos express the receptors for these molecules and hormones to obtain competence for adhesion and invasion [3]. In assisted reproductive technologies, frozen blastocyst transfer (FBT) cycles have progressively increased owing to recent improvements in vitrification techniques [4, 5]. The blastocysts cultured in vitro are transferred into the uterus after considering uterine receptivity. However, these embryos develop to the blastocyst stage without any exposure to the endometrium-derived ligands mentioned above. Therefore, even if the blastocyst is formed, implantation may fail owing to blastocyst inactivation before implantation. Thus, inducing implantation competence before FBT, such as during the recovery culture after warming, can prevent implantation failure due to lack of blastocyst activation.

Prolactin (PRL) is secreted in the endometrium during the peri-implantation period, and its synthesis extends from the late luteal phase of the menstrual cycle throughout pregnancy [6, 7]. Human embryos express the PRL receptor from the morula stage and its expression further increases during blastocyst formation [8]. Furthermore, PRL treatment from cleavage to the blastocyst stage promotes the cytoskeletal organization, epithelial–mesenchymal transition (EMT), and integrin-based focal adhesions during trophoblast migration, thereby stimulating blastocyst outgrowth. However, considering the half-life of PRL (41 min) [9] and expression pattern of the PRL receptor in embryos, PRL treatment at the blastocyst stage may be sufficient for inducing migration competence. From this hypothesis, we considered that in FBT cycles, PRL treatment after warming blastocysts cultured without PRL could improve pregnancy outcomes. However, whether post-warming culture with PRL influences outgrowth competence remains unknown. Furthermore, the optimal time for PRL treatment during recovery culture remains to be ascertained. To demonstrate the influence of PRL treatment on the implantation competence of blastocysts and optimal time for PRL treatment, we examined the expression of genes encoding ezrin–radixin–moesin (ERM) proteins, EMT, and nascent adhesion in vitrified blastocysts after PRL treatment during the recovery culture. Furthermore, we assessed whether the PRL treatment after warming promoted the outgrowth of human vitrified blastocysts.

## Methods

### Ethics statement

In total, 374 discarded, vitrified human blastocysts donated for research by consenting couples were used in this study. The study was approved by the Institutional Review Board of Kato Ladies Clinic (IRB approval number: 18–18).

### Blastocyst warming and recovery culture

Before vitrification, blastocysts were morphologically graded according to Gardner’s criteria [10]. In the present study, the blastocysts were treated with 100 ng/mL PRL, because the results from our previous study indicated that treatment with 100 ng/mL PRL from cleavage to the blastocyst stage improved blastocyst outgrowth to fibronectin [8]. The discarded blastocysts were randomly allocated to two groups, to be cultured in a medium with either 100 ng/mL PRL (*n* = 208) or without PRL (control; *n* = 166). The vitrified blastocysts were warmed using a warming kit (Kitazato Corporation, Shizuoka, Japan) according to the manufacturer’s instructions [11]. After removing the zona pellucida using acid Tyrode’s solution, the warmed blastocysts were cultured for recovery with ONESTEP medium (Nakamedical, Tokyo, Japan) for 120 min. The blastocysts in the PRL group were treated with PRL for 15–120 min during the recovery period (Additional file Fig. 1). The PRL stock solution (100 μg/mL) was stored at − 80 °C and thawed immediately before use. All embryos were cultured at 37 °C (gas phase: 5% O_2_, 6% CO_2_, and 89% N_2_) with 100% humidity in a water jacket incubator (Astec, Fukuoka, Japan).

### Blastocyst outgrowth

The proportion of adhered blastocysts and outgrowth area were examined to estimate the implantation capacity of blastocysts in vitro. The outgrowth assay was performed as described previously [8, 12]. The culture dishes were pre-coated with 10 μg/mL fibronectin (Sigma-Aldrich, Saint Louis, MO, USA) at 4 °C overnight. Next, 20 μL of ONESTEP medium without PRL was pipetted as a drop onto the slide before adding the oil overlay. The blastocysts were placed individually into the drops and cultured for 96 h for the outgrowth culture assay. Once trophoblast cells had grown outward from the blastocysts and became visible, the embryos were designated as adhesion-initiating blastocysts. The outgrowth area was measured at the end of the culture using NIS-Elements imaging software 2.0 (Nikon, Tokyo, Japan). Briefly, the outer edge of the trophoblast was selected, and the outgrowth area was automatically calculated. When the outgrowth area had decreased by more than 10% compared with the maximum area of outgrowth, the embryos were designated as degenerated. Some blastocysts were cultured using the time-lapse incubator (BZ-X800, Keyence, Osaka, Japan) to examine the migration distance of the trophoblast cells. Briefly, images were acquired every 10 min for 48–72 h after outgrowth culture. The centers of the nuclei of trophoblast cells were annotated, and the migration path was automatically traced using the BZ-X800 Analyzer software (Keyence). The distance covered by trophoblast migration for 24 h was calculated. The experiments were repeated at least 12 times.

### Gene expression analysis

A total of 70 blastocysts cultured for 12 h after the outgrowth culture were used for the gene expression analysis [PRL non-treated (control), *n* = 14; PRL 15 min, *n* = 14; PRL 30 min, *n* = 14; PRL 60 min, *n* = 14; PRL 120 min, *n* = 14]. Quantitative RT-PCR (qRT-PCR) assays were performed as previously described [8], with slight modifications. Total RNA was extracted and reverse-transcribed using Cells-to-CT Kits (Thermo Fisher Scientific, Waltham, MA, USA). Briefly, blastocysts were incubated with Lysis Solution and DNase I for 5 min, and then with Stop Solution for 2 min. The lysate was mixed with RT Master Mix and incubated for 60 min at 37 °C, followed by 5 min incubation at 95 °C in a thermal cycler. The TaqMan Gene Expression assay (Thermo Fisher Scientific) was used to quantify mRNA levels (Additional file Table 1). PCR was performed in a 20-μL reaction volume using the TaqMan Fast Universal PCR Master Mix (Thermo Fisher Scientific) and a StepOnePlus Real-time PCR System (Thermo Fisher Scientific). The expression of each target gene was normalized to that of H2A histone family member Z. Relative gene expression was quantified based on the standard curve method using StepOne software Version 2.1. Each sample was assayed in duplicate, and the experiments were repeated at least seven times.

### Statistical analyses

Statistical analyses were performed using JMP software (SAS Inc., Cary, NC, USA). Continuous parameters were compared using the Mann–Whitney U test after the analysis of distribution by the Shapiro-Wilk test. Proportion data were analyzed using Pearson’s chi-square test. Multiple linear regression analysis was used to assess the relative importance of the possible predictor variables in explaining the outgrowth area. Statistical significance was set at *P* < 0.05.

## Results

### Embryo characteristics

In total, 374 discarded human vitrified blastocysts were warmed and cultured for recovery. The characteristics of blastocysts used in this study are listed in Table 1. The age of women who donated the blastocysts, insemination method used to produce embryos, culture duration to grow until the blastocyst stage, blastocyst diameter, and morphological grade of inner cell mass and trophectoderm were comparable between the control and PRL groups. All blastocysts survived the recovery culture in both groups.

**Table 1:** Characteristics of human blastocysts used in the study

	Control	Prolactin	*P* value
No. of donated samples, n	166	208	
Donor age (year)	37.2 ± 3.7	36.9 ± 3.6	0.4942
Insemination method			
Conventional *in vitro* fertilization, *n* (%)	60 (36.1)	73 (35.1)	0.8333
Intracytoplasmic sperm injection, *n* (%)	106 (63.9)	44 (64.9)	
Culture time to the expanded blastocyst stage (h)	129.9 ± 11.4	129.7 ± 11.3	0.8380
No. of Day 5 blastocysts, *n* (%)	77 (46.4)	89 (53.6)	0.6760
No. of Day 6 blastocysts, *n* (%)	101 (48.6)	107 (51.4)	
Blastocyst diameter (μm)	181.8 ± 20.1	183.6 ± 20.0	0.3578
Morphological grade (Gardner’s criteria)			
Inner cell mass			
Grade A	26 (15.7)	35 (16.8)	0.6920
Grade B	56 (33.7)	77 (37.0)	
Grade C	84 (50.6)	96 (46.2)	
Trophectoderm			
Grade A	23 (13.9)	24 (11.5)	0.3385
Grade B	40 (24.1)	64 (30.8)	
Grade C	103 (62.1)	120 (57.7)	

Values are presented as mean ± SD or n (%)

### Influence of PRL on EMT- and focal adhesion-related genes in recovered blastocysts

**Fig. 1:**
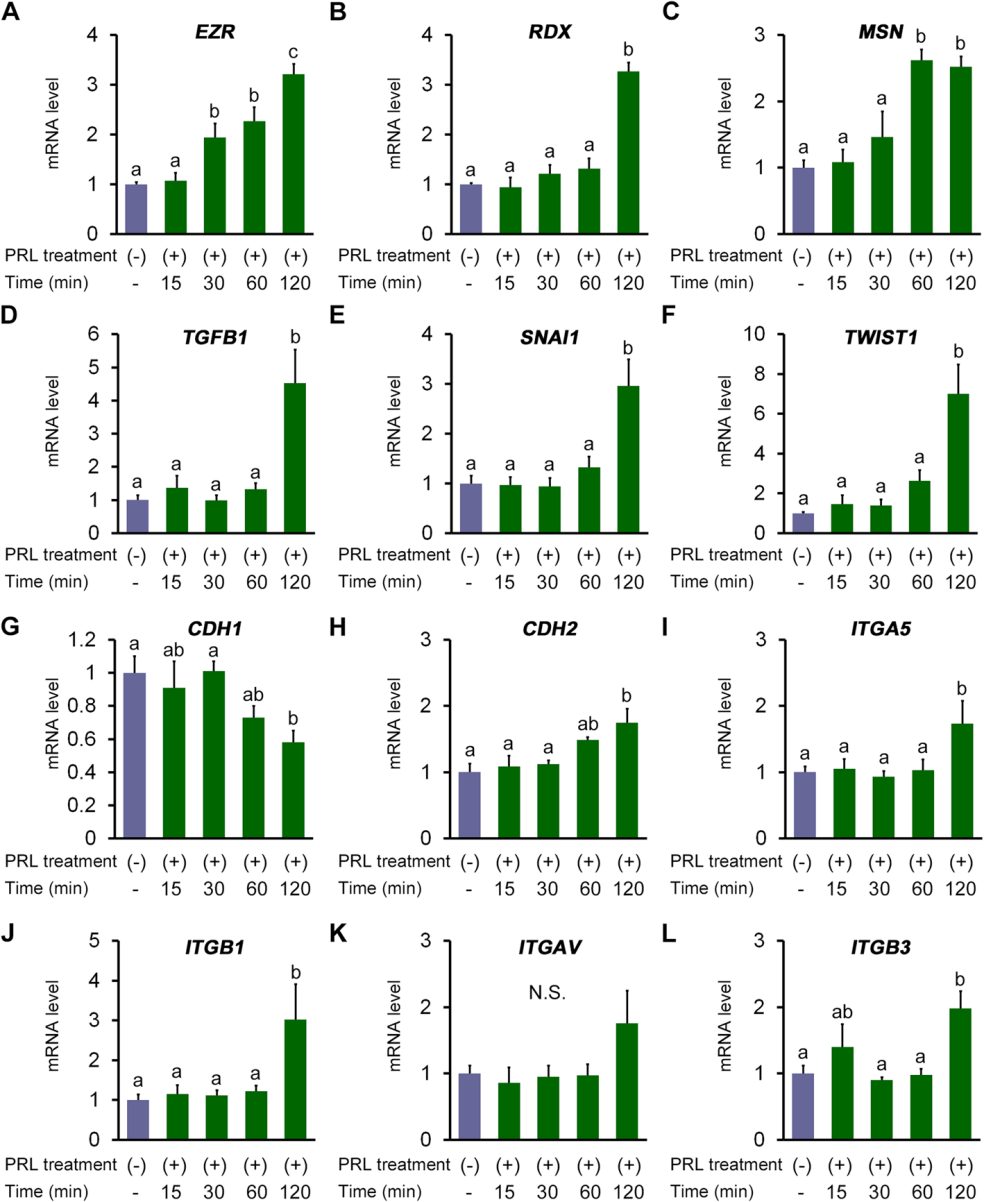
Recovery culture with prolactin (PRL) upregulates the expression of epithelial-to-mesenchymal transition (EMT)- and focal adhesion-related genes in human blastocysts. mRNA expression levels of (**A**) ezrin (*EZR*), (**B**) radixin (*RDX*), (**C**) moesin (*MSN*), (**D**) transforming growth factor beta 1 (*TGFB1*), (**E**) snail family transcriptional repressor 1 (*SNAI1*), (**F**) twist family bHLH transcription factor 1 (*TWIST1*), (**G**) cadherin 1 (*CDH1*), (**H**) cadherin 2 (*CDH2*), (**I**) integrin subunit α 5 (*ITGA5*), (**J**) integrin subunit beta 1 (*ITGB1*), (**K**) integrin subunit alpha V (*ITGAV*), and (**L**) integrin subunit β 3 (*ITGB3*) in embryos cultured on fibronectin-coated dishes for 12 h [PRL non-treated (control), *n* = 14; PRL 15 min, *n* = 14; PRL 30 min, *n* = 14; PRL 60 min, *n* = 14; PRL 120 min, *n* = 14]. The expression of each target gene was normalized to that of H2A histone family member Z. N.S., not significant. Error bars represent the standard error of the mean. ^a–c^ Different letters indicate a significant difference at *P* < 0.05

The expression of EMT- and focal adhesion-related genes was compared in blastocysts cultured with or without PRL during the recovery culture (Fig. 1A–C). The expression of ezrin (*EZR*) was stimulated by 30 min PRL treatment (*P* = 0.0038) and increased further following 120 min PRL treatment (*P* < 0.0001, Fig. 1A). The expression of radixin (*RDX*) was also promoted by 120 min PRL treatment (*P* < 0.0001, Fig. 1B). Furthermore, moesin (*MSN*) expression was upregulated by PRL treatment for 60 and 120 min (*P* < 0.0001 and *P* = 0.0002, respectively; Fig. 1C).

The effect of PRL treatment on EMT-related gene expression during recovery culture was also assessed (Fig. 1D–H). The expression of transforming growth factor β1 (*TGFB1*) (*P* < 0.0001, Fig. 1D), snail family transcriptional repressor 1 (*SNAI1*) (*P* = 0.0002, Fig. 1E), and twist family bHLH transcription factor 1 (*TWIST1*) (*P* < 0.0001, Fig. 1F) increased upon 120 min treatment with PRL. PRL-treated blastocysts also exhibited transcriptional repression of cadherin 1 (*CDH1*, *P* = 0.0168, Fig. 1G) but augmented the expression of cadherin 2 (*CDH2*, *P* = 0.0068, Fig. 1H).

Integrin-based focal adhesion-related gene expression was examined. Higher expression of integrin subunit α5 (*ITGA5*, *P* = 0.0318, Fig. 1I), integrin subunit β1(*ITGB1*, *P* = 0.0021, Fig. 1J), and integrin subunit β3 (*ITGB3*, *P* = 0.0044, Fig. 1L) was observed after 120 min PRL treatment than in the non- and shorter-treatment groups. Although the expression of integrin subunit αV (*ITGAV*) was higher after 120 min PRL treatment than in the other groups, the increase was statistically insignificant (Fig. 1K).

### Influence of PRL treatment during recovery culture on the blastocyst outgrowth

**Fig. 2:**
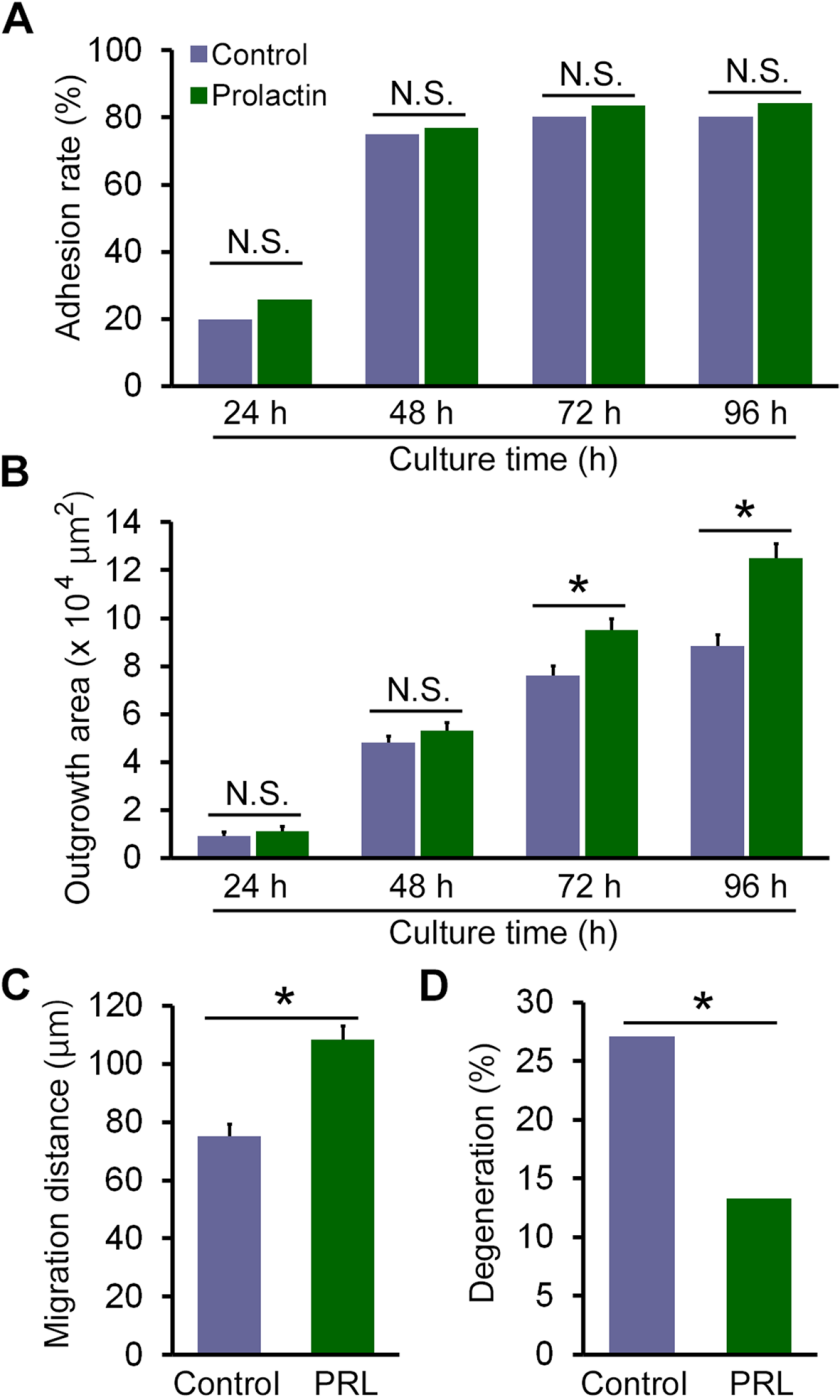
Prolactin (PRL) treatment during recovery culture promotes trophoblast migration and reduces outgrowth degeneration. **A** Comparison of blastocyst adhesion rates to the fibronectin-coated dishes between the control and PRL-treated blastocysts (control, *n* = 152; PRL 120 min, *n* = 152); **B** comparison of outgrowth area after 96 h of outgrowth culture between the control and PRL-treated blastocysts; **C** migration distance of trophoblast cells at 48–72 h after outgrowth culture; **D** rates of outgrowth degeneration after 96 h outgrowth culture. N.S., not significant. Error bars represent the standard error of the mean. ^*^Significant difference at *P* < 0.05

Blastocyst competence for outgrowth and adhesion to the fibronectin-coated dishes was compared between blastocysts cultured without or with PRL for 120 min (control, *n* = 152; PRL 120 min, *n* = 152). Recovery culture with PRL did not alter the rate of blastocyst adhesion to fibronectin-coated dishes 96 h after the outgrowth culture assay (Fig. 2A). However, the outgrowth area at 72 and 96 h was significantly larger in the PRL group than in the control group (*P* = 0.0019, *P* < 0.0001, respectively; Fig. 2B). The multiple linear regression analyses also revealed significantly larger outgrowth area in PRL-treated blastocysts (Additional file Table 2). Additionally, the migration distance of trophoblast cells was significantly higher in the PRL group than in the control group (*P* < 0.0001, Fig. 2C). Furthermore, the degeneration rate was significantly lower in the PRL group than in the control group (*P* = 0.0065, Fig. 2D).

### Stratified outcomes of outgrowth assay according to blastocyst characteristics

**Fig. 3:**
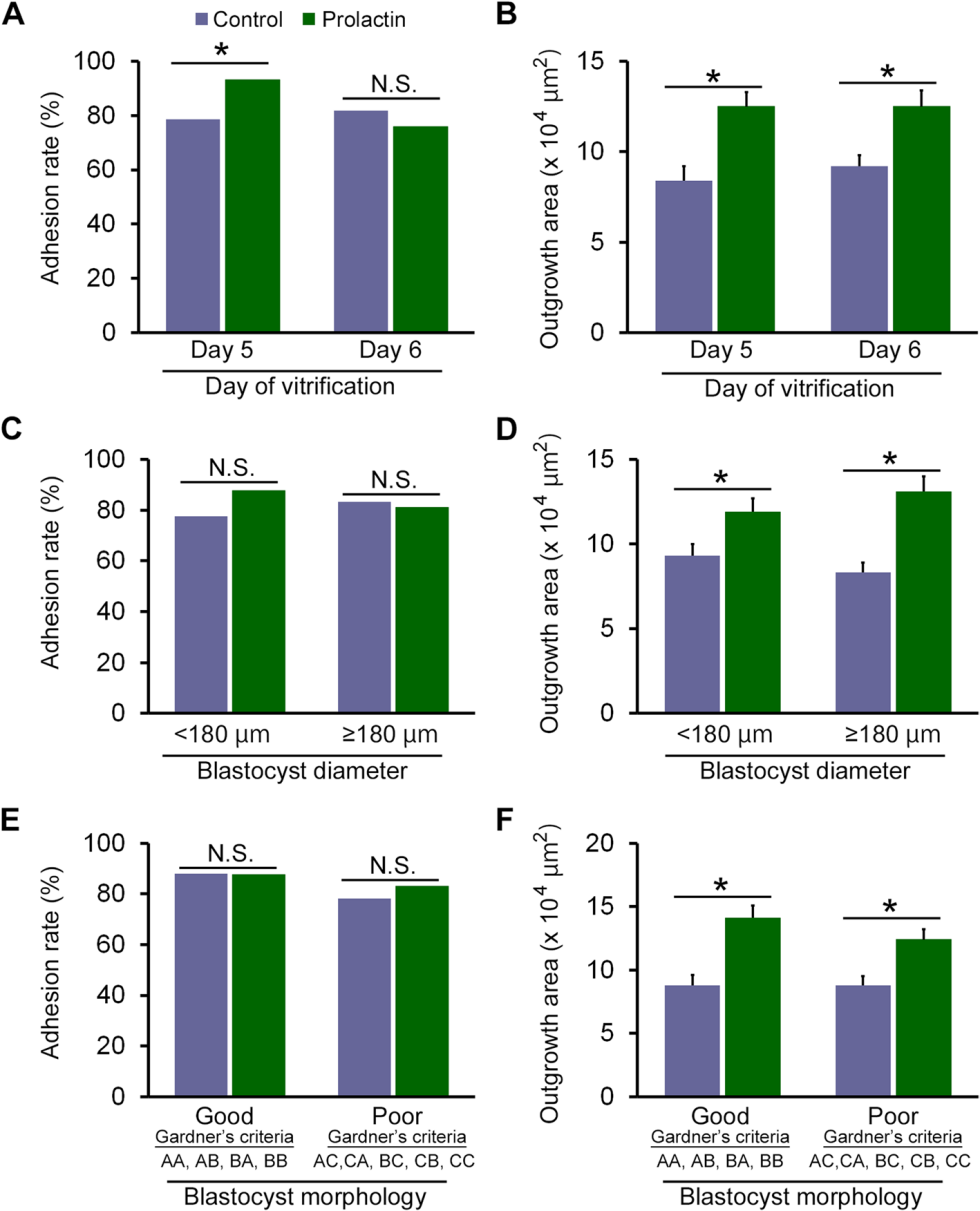
Stratified blastocyst outgrowth according to blastocyst characteristics. The blastocyst adhesion rate and outgrowth area stratified by blastocyst vitrification day (Day 5 vs. Day 6; **a** and **b**, respectively), diameter (< 180 μm vs. ≥180 μm; **c** and **d**, respectively), or morphology (Good [Gardner’s criteria: AA, AB, and BA] vs. Poor [Gardner’s criteria: AC, CA, BC, CB, and CC]; e and f, respectively). N.S., not significant. Error bars represent the standard error of the mean. ^*^Significant difference at *P* < 0.05

Blastocyst outgrowth assay results were stratified according to the day of blastocyst vitrification (Fig. 3A and B). When the blastocysts were vitrified on day 5, the blastocyst adhesion rate was significantly increased by recovery culture with PRL (*P* = 0.0120, Fig. 3A). However, the adhesion rates were comparable between the groups when the blastocysts were vitrified on day 6. The outgrowth area significantly increased after the recovery culture with PRL, regardless of the day of blastocyst vitrification (day 5, *P* = 0.0007; day 6, *P* = 0.0002: Fig. 3B).

We analyzed the stratified outcomes of the outgrowth assay according to the blastocyst diameter, < 180 μm or ≥ 180 μm (Fig. 3C and D). The adhesion rates were comparable between the groups, regardless of the blastocyst diameter (Fig. 3C). However, the outgrowth area was significantly larger in the PRL group than in the control group in both diameter stratifications (*P* = 0.0217, *P* < 0.0001, respectively; Fig. 3D).

When stratified according to blastocyst morphology using Gardner’s criteria (good quality: AA, AB, and BA; poor quality: AC, CA, BC, CB, and CC), adhesion rates were comparable between the groups, regardless of the blastocyst morphology (Fig. 3E). However, the outgrowth area was significantly larger in the PRL group than in the control group in both stratifications (*P* < 0.0001, *P* = 0.0010, respectively; Fig. 3F).

## Discussion

Here, we report that 120 min PRL treatment during recovery culture promoted trophoblast outgrowth in vitrified human blastocysts by upregulating the expression of EMT- and focal adhesion-related genes. Furthermore, our results revealed a more beneficial effect of PRL treatment on blastocyst adhesion and outgrowth when the blastocysts were vitrified on day 5 than when they were vitrified on day 6.

EMT, a developmentally conserved trans-differentiation process, is essential for trophoblast invasion into the endometrium. The trophoblast cells lose their apicobasal polarity and can migrate and invade, which are mesenchymal traits [13]. We demonstrated that recovery culture with PRL stimulated the mRNA expression of *EZR*, *RDX*, and *MSN*, which regulate cell adhesion and invasion by controlling actin reorganization during EMT [14, 15]. Furthermore, the expression of the EMT-inducer gene *TGFB1* [16–18] was promoted by PRL treatment. PRL treatment also upregulated the expression of *SNAI1* and *TWIST1*, which are necessary to initiate EMT [19, 20]. *SNAI1* and *TWIST1* directly modulate the expression of EMT-related core genes and alter the cellular phenotype [13, 19]. PRL-treated blastocysts exhibited augmented expression of *CDH2* but transcriptional repression of *CDH1*. Therefore, PRL treatment during recovery culture may induce EMT by stimulating the expression of ERM proteins and EMT-related genes. Our data showed that EMT can be induced in blastocysts when subjected to 120 min recovery culture with PRL. In FBT, the frozen embryos are cultured for 2–3 h after warming to confirm their viability and recovery. Thus, our results suggest that 120 min PRL treatment during recovery culture can enhance blastocyst viability and recovery in clinical settings.

Human trophoblast cells express several integrins [21, 22] and adhere to fibronectin through the integrin heterodimers α5β1 [23] and αvβ3 [24], which mediate nascent adhesion during the formation of lamellipodial protrusions [25, 26]. In this study, the increased expression of integrin-based focal adhesion-related genes *ITGA5*, *ITGB1*, and *ITGB3* was observed after 120 min PRL treatment. Therefore, competence for trophoblast adhesion and migration may be stimulated by recovery culture in the presence of PRL. Furthermore, our results demonstrated that the outgrowth area of blastocysts was significantly increased by 120 min PRL treatment, although the blastocyst adhesion rate was not altered. The migration distance of trophoblast cells significantly increased after PRL treatment, suggesting that PRL-stimulated trophoblast migration and integrin-based focal adhesion led to increased outgrowth area.

The incidence of outgrowth degeneration was lower in PRL-treated blastocysts than in non-treated blastocysts (from 27 to 13%) (Additional file Video 1). EMT allows a polarized epithelial cell to undergo multiple biochemical changes to eventually assume a mesenchymal cell phenotype with enhanced migratory capacity, invasiveness, proliferative action, increased resistance to apoptosis, and increased production of extracellular matrix components [27–29]. Therefore, the recovery culture in presence of PRL may reduce apoptosis of migrating trophoblast cells and strengthen blastocyst adhesion to the endometrium.

In our study, PRL treatment was more effective for the blastocysts vitrified on day 5 than those vitrified on day 6. This result can be explained by our previous report that the expression level of the PRL receptor in the human blastocysts is significantly impacted by the culture duration required for embryos to reach the blastocyst stage, diameter, and morphology [8]. However, the effectiveness of PRL treatment was not affected by the blastocyst diameter or morphology as the treatment promoted its outgrowth in both stratified groups. The diameters of blastocysts used in the present study were measured immediately before vitrification; the diameters of those vitrified on day 5 are likely to be smaller than those vitrified on day 6. Therefore, if the diameter was measured at the same time, the results can vary from this study.

This study has several limitations. First, the outgrowth assay used in this study evaluated blastocyst adherence and migration on a two-dimensional surface, which is not a definitive measure of invasion of trophoblast cells. Therefore, modified invasion assays should be conducted for more definitive results. Moreover, results may vary between in vivo and in vitro conditions because the blastocysts were not exposed to other growth factors, cytokines, and epigenetic regulators derived from the endometrium. Furthermore, we only evaluated the effects of 100 ng/mL PRL on outgrowth and gene expression. The half-life of PRL is 41 min [9]; therefore, the effects of other concentrations or other preparation methods on the blastocyst capacity for implantation remain unclear. In addition, future studies should focus on the effect of PRL treatment on the decrease of outgrowth degeneration. Although we demonstrated the positive effects of PRL treatment during the recovery culture, these results may only be observed when the zona pellucida is removed before the recovery culture. The effects of PRL treatment on the zona pellucida-enclosed blastocysts should be also examined.

## Conclusions

Our findings indicate that 120 min PRL treatment promoted trophoblast outgrowth in vitrified human blastocysts by upregulating the expression of EMT- and focal adhesion-related genes. These results suggest that recovery culture with PRL prior to FBT improves pregnancy outcomes after FBT. Therefore, further clinical studies are needed to explore the clinical efficacy of PRL treatment during the recovery culture to improve outcomes after FBT.

## Supplementary Information

**Additional file 1: Figure 1.** Outline of prolactin treatment during the blastocyst recovery culture

**Additional file 2: Table 1.** TaqMan Gene Expression Assay used for quantification of mRNA expression. **Table 2.** Multiple linear regression analysis coefficients for outgrowth area.

**Additional file 3: Video 1.** Degeneration of blastocyst outgrowth.

## Data Availability

The datasets used and/or analyzed during the present study are available from the corresponding author on reasonable request.

## Abbreviations

PRL: Prolactin
EMT: Epithelial-to-mesenchymal transition
FBT: Frozen blastocyst transfer
ERM: Ezrin-radixin-moesin [proteins]
