# Supplementary figures and images for: Post-warming culture of human vitrified blastocysts with prolactin improves trophoblast outgrowth
Source: Reprod Biol Endocrinol. 2023 Jan 18;21:6. doi: 10.1186/s12958-023-01062-0 (PMC9847091; doi:10.1186/s12958-023-01062-0)

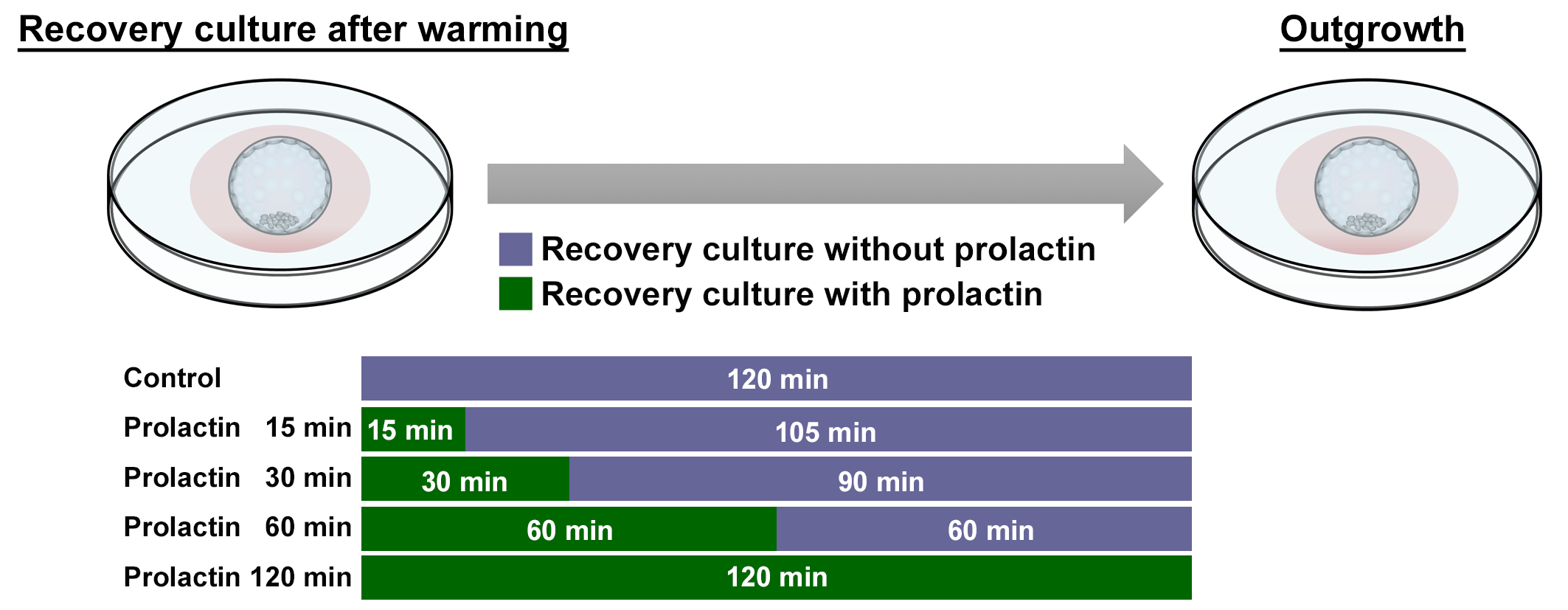

Supplement: Supplementary file 1 — Additional file 1: Figure 1. Outline of prolactin treatment during the blastocyst recovery culture [file 12958_2023_1062_MOESM1_ESM.tif]
