# Supplementary material for: Post-warming culture of human vitrified blastocysts with prolactin improves trophoblast outgrowth
Source: Reprod Biol Endocrinol. 2023 Jan 18;21:6. doi: 10.1186/s12958-023-01062-0 (PMC9847091; doi:10.1186/s12958-023-01062-0)
Supplement: Supplementary file 2 — Additional file 2: Table 1. TaqMan Gene Expression Assay used for quantification of mRNA expression. Table 2. Multiple linear regression analysis coefficients for outgrowth area. [file 12958_2023_1062_MOESM2_ESM.docx]

**Additional files**

**Post-warming culture of human vitrified blastocysts with prolactin improves trophoblast outgrowth**

**Tables**

**Table 1. TaqMan Gene Expression assay used for the quantification of mRNA levels**

| **Gene name** | **Gene symbol** | **Assay ID** |
| --- | --- | --- |
| Ezrin | *EZR* | Hs00931646_m1 |
| Radixin | *RDX* | Hs00988414_g1 |
| Moesin | *MSN* | Hs00741306_mH |
| Transforming growth factor beta 1 | *TGFB1* | Hs00998133_m1 |
| Snail family transcriptional repressor 1 | *SNAI1* | Hs00195591_m1 |
| Twist family bHLH transcription factor 1 | *TWIST1* | Hs01675818_s1 |
| Cadherin 1 | *CDH1* | Hs01023895_m1 |
| Cadherin 2 | *CDH2* | Hs00983056_m1 |
| Integrin subunit alpha 5 | *ITGA5* | Hs01547673_m1 |
| Integrin subunit beta 1 | *ITGB1* | Hs01127536_m1 |
| Integrin subunit alpha V | *ITGAV* | Hs00233808_m1 |
| Integrin subunit beta 3 | *ITGB3* | Hs01001469_m1 |

**Table 2. Multiple linear regression analysis coefficients for outgrowth area**

|  | Regression coefficient | 95% confidence intervals | | Standard error | *t* value | *P* value |
| --- | --- | --- | --- | --- | --- | --- |
|  |  | Lower | Upper |  |  |  |
| Donor age | −4067.7 | −6090.3 | −2045.1 | 1026.8 | −3.96 | < 0.0001 |
| Time required for the embryos reach the blastocyst stage | 411.3 | −384.0 | 1206.5 | 403.7 | 1.02 | 0.3094 |
| Blastocyst diameter | −77.4 | −455.4 | 300.6 | 191.9 | −0.40 | 0.6870 |
| Inner cell mass |  |  |  |  |  |  |
| Grade A | Reference | – | – | – | – | – |
| Grade B | −7025.5 | −18418.8 | 4367.9 | 5783.8 | −1.21 | 0.2257 |
| Grade C | −6079.8 | −17291.4 | 5131.9 | 5691.6 | −1.07 | 0.2865 |
| Trophectoderm |  |  |  |  |  |  |
| Grade A | Reference | – | – | – | – | – |
| Grade B | 2894.3 | −9992.7 | 15781.3 | 6542.1 | 0.44 | 0.6586 |
| Grade C | −1760.7 | −13705.2 | 10183.8 | 6063.6 | −0.29 | 0.7718 |
| Medium for recovery culture |  |  |  |  |  |  |
| Control | Reference | – | – | – | – | – |
| Prolactin | 18158.1 | 10636.7 | 25679.5 | 3818.3 | 4.76 | < 0.0001 |
